# Effects of Intensive Glycemic Control on Serum Exosome miR-126-3p and miR-125b-1-3p Levels and Wound Healing in Patients with Diabetic Ulcers

**DOI:** 10.1155/2023/2523245

**Published:** 2023-01-30

**Authors:** Lin Wang, Aitian Zheng, Na Zeng, Zheng Li, Lizhu Tang, Caidan Long, Biaoliang Wu

**Affiliations:** ^1^Department of Endocrinology, The Affiliated Hospital of Youjiang Medical University for Nationalities, Baise, Guangxi, China; ^2^Department of Endocrinology, Pingshan District People's Hospital, Pingshan General Hospital of Southern Medical University, Shenzhen, Guangdong, China

## Abstract

**Objective:**

Intensive glycemic control and exosomal miRNAs have both been reported to improve wound repair in diabetic ulcers. In this study, we aimed to investigate the effects of intensive glycemic control on serum exosome microRNA-126-3p (miR-126-3p), microRNA-125b-1-3p (miR-125b-1-3p), and wound healing in patients with diabetic ulcers.

**Methods:**

Herein, 45 diabetic patients with an ulcer, aged 35–75 years old, were randomly assigned to the intensive glycemic control group (*n* = 21) and the conventional glycemic control group (*n* = 24). Serum exosomes were extracted in the laboratory and assessed by Western blotting, transmission electron microscopy, and nanoparticle tracking analysis. The expression of miR-126-3p and miR-125b-1-3p was validated using quantitative real-time polymerase chain reaction. The wound healing of each diabetic ulcer patient was measured and imaged; additionally, clinical and follow-up data were collected. Finally, the clinical and laboratory data were combined for statistical analysis.

**Results:**

Intensive glycemic control was significantly more conducive to wound healing and infection control than conventional glycemic control (*P* < 0.05). Serum exosomal miR-126-3p was negatively correlated with fasting plasma glucose levels (*r* = 0.34, *P* < 0.05) and positively associated with the wound healing rate (*r* = 0.45, *P* < 0.01). The level of miR-126-3p in the intensive glycemic control group was significantly higher than that in the conventional glycemic control group (*P* < 0.01). Serum exosomal miR-125b-1-3p was not correlated with blood glucose levels (*r* = 0.03, *P* > 0.05) and was positively associated with the wound healing rate (*r* = 0.33, *P* < 0.05). No significant difference was observed in the level of miR-125b-1-3p between the intensive and conventional glycemic control groups. Regarding the prognosis of diabetic ulcers, the intensive glycemic control group was better than the conventional group (*Z* = −2.02, *P* < 0.05).

**Conclusion:**

Serum exosome (miR-125b-1-3p and miR-126-3p) levels are correlated with wound healing in diabetic ulcers. Intensive glycemic control increases the serum exosomal miR-126-3p level, which might be one of the mechanisms that promotes wound healing in diabetic ulcers.

## 1. Introduction

Diabetic ulcer (DU) is a serious complication of diabetes and an important cause of disability and death in patients with diabetes. The disease is more common in the feet of patients, so it is also called diabetic foot. The predisposing factors of DU are related to persistent wound inflammation and prolonged nonhealing of skin wounds due to hyperglycemia. Persistent hyperglycemia further leads to peripheral neuropathy, vascular endothelial injury, and skin ulceration secondary to arterial vascular occlusion [1, 2]. Therefore, the control of glucose and lipid metabolism disorders caused by diabetes is the basis for the treatment of DU. Intensive glycemic control emerges from this, and it plays an active role in the 4 steps of wound healing: hemostasis, inflammation, proliferation, and remodeling [3, 4]. Intensive glycemic control can improve diabetic neuropathy, reduce the incidence of wounds, and reduce the amputation rate of the diabetic foot, which in turn is conducive to the repair and healing of diabetic ulcers [5].

Wound healing is a complex, dynamic process involving multiple cytokines and growth factors and the synergistic cooperation between different cells. A growing body of evidence suggests that miRNA-laden exosomes are becoming key regulators in the wound healing process. For example, miRNA-21-5p has been reported to promote angiogenesis by activating the MAPK, PI3K/Akt, and ERK1/2 pathways [6, 7]. The miR-21-3p has been reported to enhance the proliferation and migration of fibroblasts, promote endothelial cell angiogenesis, and accelerate wound healing [7, 8]. Of course, there are also some miRNAs that inhibit wound repair, such as miR-24-3p, miRNA-195-5p, and miR-205-5p [9, 10]. Exosomal miRNAs have the advantages of low rejection and good histocompatibility, so they can be used as effective targets and potential drugs for the treatment of diabetic foot ulcers [11, 12].

We previously discovered that miR-125b-1-3p and miR-126-3p are miRNAs that have potential effects on wound repair; these miRNAs reduce wound inflammation and promote vascular endothelial cell proliferation [13–15]. However, no studies have explored the effect of intensive glycemic control on serum exosomal miR-125b-1-3p and miR-126-3p. This study investigated the effect of intensive glycemic control on serum exosome miR-126-3p and miR-125b-1-3p levels and wound healing in patients with diabetes mellitus and ulcer wounds.

## 2. Materials and Methods

### 2.1. Ethics Statement

The collection of all serum samples and wound tissue in this experiment was approved by the participants, and they were made aware of the content and methods of the study. The study was approved by the Ethics Committee of the Affiliated Hospital of Youjiang Medical College for Nationalities, and the ethics number is 2019031501.

### 2.2. Research Subjects, Randomization, and Treatment Options

This study included 45 patients with diabetic ulcers and 10 healthy subjects. The samples were obtained from all individuals who visited the Affiliated Hospital of Youjiang Medical College for Nationalities from June 2019 to February 2021. The diagnostic criteria for diabetes were in accordance with the standards of the World Health Organization (1999) [16]. Cases of pregnancy, malignant tumors, other serious metabolic diseases, cardiovascular and cerebrovascular diseases, psychiatric diseases, immunodeficiency diseases, recurrent hypoglycemia during treatment, and unwillingness to cooperate were excluded from this study. Moreover, the cases in the experimental group all met the screening criteria for the first skin ulcer wound on the basis of pre-existing diabetes. The patients took metformin, acarbose, and sulfonylurea hypoglycemic drugs before admission without intensive treatment with insulin. Some patients did not even use the drugs outside the hospital and used insulin to control blood sugar after admission. Patients had both acute wounds and chronic wounds over 4 weeks. The wound area was 0.7 cm^2^–84 cm^2^. The degree of wounds included Wagner's classification grades 1–3. Patients with diabetic ulcer wounds were randomized after admission. The serum samples of healthy subjects were used as a blank control group to obtain the relative expression of serum exosomal miRNAs in the experimental group. The healthy physical examiner was in good health before, and no liver, kidney, or lung lesions were found in this physical examination. There were no metabolic diseases, such as diabetes and there were no acute or chronic wounds.

According to the randomized trials, the patients were divided into the following two groups: 21 cases in the intensive glycemic control group and 24 cases in the conventional glycemic control group. The conventional glycemic control target was fasting blood glucose (FBG) of 4.4–7.0 mmol/L and postprandial blood glucose (2 h PBG) of ≤10.0 mmol/L after two hours [17]. The goal of intensive glycemic control was FBG of 4.4–6.1 mmol/L and 2 h PBG of 4.4–8.0 mmol/L [18]. The standard for the intensive glycemic control group was that the goal of intensive control must be achieved for more than 7 consecutive days.

Treatment was oral hypoglycemic drugs or combined insulin, and adjuvant therapy was to control blood pressure, blood lipids, and secondary preventive drugs for cardiovascular disease. Daily debridement with saline and intravenous antibiotics was performed if necessary. Fingertip blood glucose was monitored 7 times a day, specifically before meals, 2 hours after meals, and before bedtime.

### 2.3. General Information, Clinical Data, and Laboratory Data

The patient's name, age, sex, hospital number, telephone number, home address, occupation, tobacco and alcohol habits, family history, course of illness, number of episodes, treatment plan, concomitant diseases, and other information were recorded. Patient height, weight, blood glucose (HbA1c, fasting blood glucose, and postprandial blood glucose), blood pressure, blood lipids, liver and kidney function, routine blood tests, infection indicators, wound microbial culture, electromyography, and other indicators were collected. The serum samples of patients with diabetic ulcers were collected on day 1 of admission and day 10 of standardized treatment. The return visit dates were 2 weeks, 4 weeks, 6 weeks, 8 weeks, and 12 weeks after enrollment. Follow-up visits were made once a month until the wound healed or the endpoint event occurred.

### 2.4. Exosome Extraction and Identification

The cell debris was removed by centrifugation of the serum sample at 3000 × *g* and 4°C for 15 min. EVs were extracted using an ExoEasy Maxi Kit (QIAGEN, GmbH, Hilden, Germany) and the following methods to identify exosomes. The protein concentration was determined by a BCA Protein Assay Kit (Cat#23225, Thermo Scientific, USA). Then, the samples were separated using 5% SDS‒PAGE, and the proteins were transferred to a membrane (Millipore Corporation, USA, Item No. IPVH00010). Next, the membrane was blocked with nonfat dry milk and probed with primary and secondary antibodies to detect the exosomal surface markers CD9, TSG101, and CD63. For transmission electron microscopy (TEM), we collected 20 *μ*L samples and placed them on a carbon film copper mesh for 3–5 min, followed by the addition of 2% phosphotungstic acid. This method was applied to analyze the morphology of exosomes and collect images. For nanoparticle tracking analysis (NTA), ZetaView PMX 110 (particle-matrix) and ZetaView 8.04.02 softwares were used to measure the size and concentration of the exosomes. Origin 8.5 software was used to draw the particle size and plot the concentration distribution.

### 2.5. RNA Extraction, Reverse Transcription, and Real-Time Quantitative PCR Detection System (qRT-PCR) Technology

The total RNA was extracted using TRIzol (Invitrogen) reagent according to the manufacturer's protocol. RNA was reverse-transcribed into complementary DNA using the miRcute miRNA first-strand cDNA synthesis kit (KR211, Tiangen, Beijing, China). SYBR Green (FP411, Tiangen, Beijing, China) and a Bio-Rad CFX96 Real-Time System (Bio-Rad) were applied to quantify PCR amplification. U6 was used as an internal reference, and the significance of changes in mRNA was calculated using the 2^−∆∆*t*^ by Cq value. The miRNA tailing method for one-way primer sequence design is shown in Table 1.

### 2.6. Recording of the Area of the Patient's Ulcers and Calculation of the Healing Rate of Ulcers

Medical rotating rulers were used by trained technicians to measure the wound area, take pictures, and analyze the data using ImageJ software. The formula for calculating the diabetic ulcer area was as follows: wound healing rate = (original wound area − unhealed wound area)/original wound area × 100%.

### 2.7. Statistical Methods

The SPSS 26.0 and GraphPad Prism 7 software were used for statistical analysis and data visualization. The measurement data that conformed to the normal distribution are represented by the mean ± standard deviation ($\bar{x}$ ± sd), and those that did not conform to the normal distribution are represented by (*M* (*Q*_*L*_ − *Q*_*u*_)). For intragroup comparisons, a paired *t*-test was used for normally distributed data, while the Wilcoxon rank-sum test was used for nonnormally distributed data. For comparisons between groups, the independent-sample*t*-test was used for normal distributions, and the Mann–Whitney *U* test was utilized for nonnormal distributions. Normally distributed data were evaluated by Pearson's correlation analysis. Pearson's chi-square test was applied to compare enumeration data. *P* < 0.05 indicated a significant difference.

## 3. Results

### 3.1. Baseline Characteristics of the Two Groups

No statistically significant differences were detected in sex, age, body mass index (BMI), blood sugar, glycosylated hemoglobin A1c, blood pressure, protein, blood lipids, uric acid, infection indicators, or other baseline data between the intensive and conventional glycemic control groups before treatment (all *P* > 0.05, Table 2).

### 3.2. Comparison of Clinical Data within and between Groups

**Figure 1:**
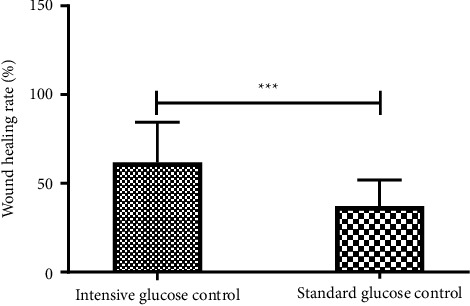
Comparison of wound healing rate between the two groups, ^*∗∗∗*^*P*  <  0.001.

In both groups, the white blood cell (WBC) count, neutrophil percentage, and ulcer area after treatment were significantly reduced compared to those before treatment (all *P* < 0.05, Table 3). The comparison between the two groups revealed that the WBC value of the conventional glycemic control group decreased, and the wound healing rate was higher than that of the conventional glycemic control group (all *P* < 0.05, Figure 1).

### 3.3. Serum Exosomes

**Figure 2:**
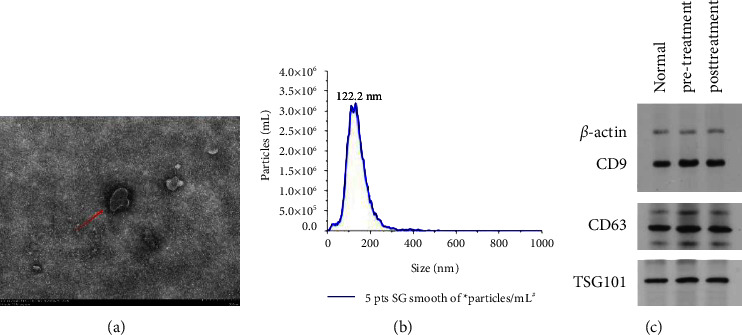
Exosomes identification results. Under the TEM, the double-layer membrane structure can be identified, which is an elliptical vesicle with a diameter between 30 and 150 nm (a). NTA shows that the peak size of the particle size is around 122.2 nm, and the concentration is 3.0 × 10^6^/mL. The particle size of this size accounts for 97.8% of the ratio (b). In patients with diabetic ulcers, the protein bands of serum exosome surface markers CD63, CD9, and TSG101 were all positive before and after treatment (c).

Three methods were used to prove the successful extraction of exosomes based on morphology, size, and surface marker proteins. WB, TEM, and NTA were utilized to detect CD9, TSG101, and CD63 surface signaling proteins (Figure 2).

### 3.4. Serum Exosome miRNAs, Blood Sugar, and the Wound Healing Rate

**Figure 3:**
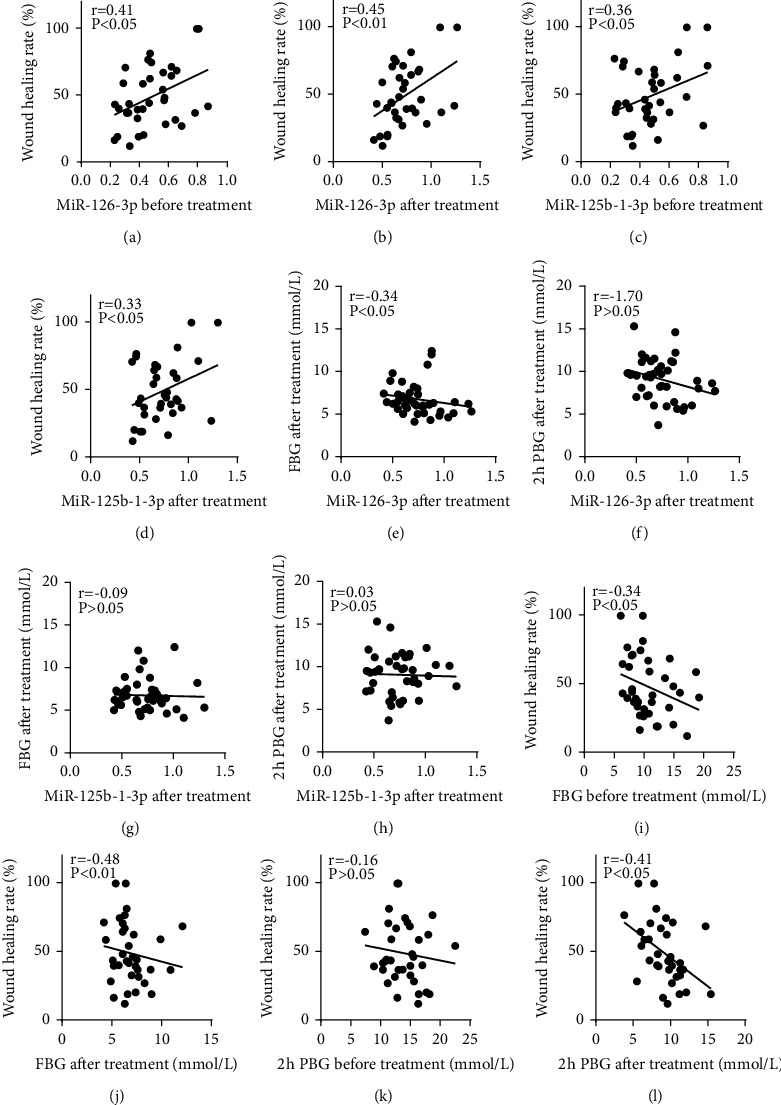
Correlation analysis of serum exosome microRNAs with wound healing rate and blood glucose. The levels of serum exosome miR-126-3p and miR-125b-1-3p before and after glycaemic control are positively correlated with wound healing rate, *P*  <  0.05 (a–d). Serum exosome miR-126-3p levels after treatment are negatively correlated with fasting blood glucose (FBG) levels after treatment, *P* < 0.05 (e). Serum exosome miR-126-3p level after treatment has no significant correlation with 2-hour postprandial blood glucose (2 h PBG) levels after treatment, *P* > 0.05 (f). Serum exosome miR-125b-1-3p levels after treatment have no significant correlation with after treatment FBG (g) and 2 h PBG (h), all *P* > 0.05. Fasting blood glucose before (i) and after (j) treatment is negatively correlated with wound healing rate, all *P* < 0.05. There is no obvious correlation between 2 h PBG and wound healing rate before treatment, *P* > 0.05 (k). 2 h PBG is negatively correlated with wound healing rate after treatment, *P*  <  0.05 (l).

The relative expression levels of miR-126-3p and miR-125b-1-3p in patient serum exosomes were obtained through qRT-PCR, and the correlations with blood glucose levels and wound healing rates were analyzed (Figure 3). Next, serum exosome (miR-126-3p and miR-125b-1-3p) levels were positively correlated with wound healing. Serum exosomal miR-126-3p levels were negatively correlated with FBG, and wound healing was negatively correlated with blood sugar.

### 3.5. Comparison of the Relative Expression Levels of Serum Exosomal miR-126-3p and miR-125b-1-3p between the Two Groups

**Figure 4:**
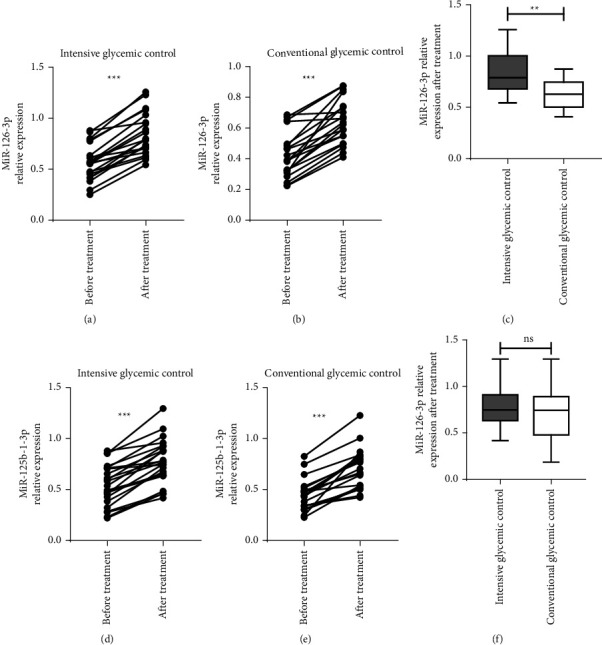
The relative expression levels of serum exosome miR-126-3p and miR-125b-1-3p before and after treatment in the two groups, and the differences in expression levels between groups. The level of serum exosome miR-126-3p in the intensive glycemic control group (a) and the conventional glycemic control group (b) increased after treatment, *P* < 0.001. After treatment, the serum exosome miR-126-3p level in the intensive glycemic control group was higher than that of the conventional glycemic control group, *P*  <  0.01 (c). The level of serum exosome miR-125b-1-3p increased in the intensive glycemic control group (d) and the conventional glycemic control group (e) after treatment, and the differences are statistically significant, all *P* < 0.001. There was no significant difference in serum exosome miR-125b-1-3p level in the intensive glycemic control group after further comparison, *P*  >  0.05 (f). ^*∗∗*^*P*  <  0.01, ^*∗∗∗*^*P*  <  0.001.

Serum miR-126-3p and miR-125b-1-3p levels increased in the two groups after treatment, and the serum exosome miR-126-3p level in the intensive glycemic control group was higher than that in the conventional glycemic control group (Figure 4).

### 3.6. Prognosis of the Two Groups

**Figure 5:**
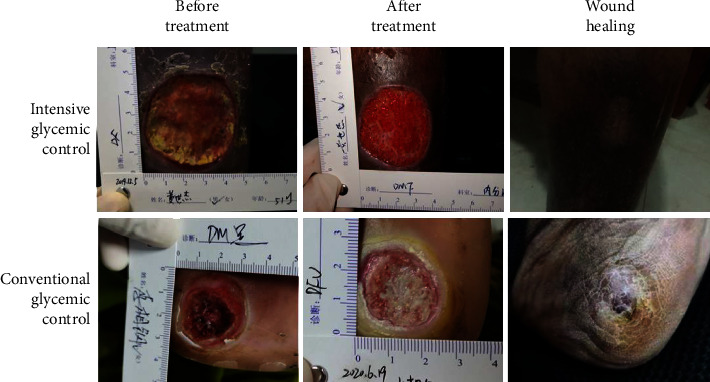
Diabetic ulcers development map. The time points were after the first debridement, when the second serum was collected, and when healed.

We grouped the patients based on prognosis, and 4 patients were lost to follow-up. The patients whose ulcers healed within 3 months were classified as having a good prognosis, and patients with chronic wounds, wound recurrences, amputations, or deaths were classified as having a poor prognosis. The ulcer prognosis of patients in the intensive glycemic control group was better than that in the conventional glycemic control group (*P* < 0.05; Table 4, Figure 5).

### 3.7. Analysis of Survival Curves: Diabetic Ulcer and Exosome miRNA Levels

**Figure 6:**
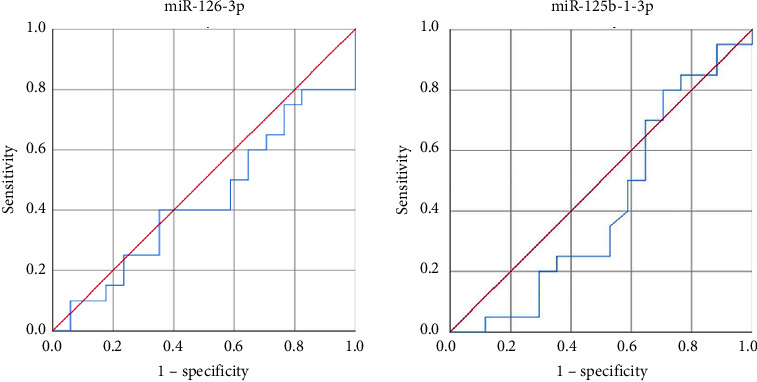
The relative expression levels of exosome miR-126-3p and miR-125b-1-3p to plot the ROC curve of wound prognosis (good/bad).

The relative expression levels of exosomal miR-126-3p and miR-125b-1-3p were plotted to obtain the receiver operating characteristic (ROC) curve of wound prognosis (good/bad) (Figure 6). We observed that the area under the ROC curve of miR-126-3p and miR-125b-1-3p was 0.432 and 0.416, and the *P* values were 0.483 and 0.385, respectively. Therefore, the relative expression levels of miR-126-3p and miR-125b-1-3p could not be used as indicators for evaluating the prognosis of diabetic ulcers.

## 4. Discussion

Through clinical observation and regular follow-up of blood glucose and wounds outside the hospital, this study found that the intensive glycemic control group healed faster and that the prognosis was better than that of the conventional glycemic control group (Table 4). This conclusion is consistent with the study by Xiang et al. [19]. These researchers showed that maintaining HbA1c levels between 7.0% and 8.0% promotes the healing of diabetic ulcers, and patients with better blood sugar on admission heal rapidly. Previous studies have confirmed that hyperglycemia is not conducive to ulcer healing at any stage, as it weakens the phagocytosis and chemotaxis of neutrophils, hinders the synthesis of collagen, impedes the migration of keratinocytes, and induces endothelial progenitor cell dysfunction and polarization of M2 macrophages [20–23]. However, only a few studies have used experimental data to illustrate the positive effect of intensive glycemic control on diabetic ulcers. This is mainly related to the difficulty of blood glucose management and the difficulty of obtaining data such as blood glucose values and wound area.

The healing process of diabetic ulcer wounds includes hemostasis, inflammation, proliferation, and remodeling [4]. In the early stages of inflammation, tumor necrosis factor *α*, interleukin-1 (IL-1), IL-6, etc., can recruit monocytes, T cells, and eosinophils, among others, to participate in the inflammatory response [24]. This study compared the clinical data and demonstrated that the intensive glycemic control group had an inflammation index that was reduced more than that of the conventional glycemic control group, which is beneficial for shortening the inflammatory response time. Patients with intensive glycemic control had a higher wound healing rate than those with conventional glycemic control (Table 3 and Figure 1), which is consistent with the study by Rastogi et al. [25]. During continued return visits, the patients were instructed to regulate their blood sugar outside the hospital, change the dressings, and observe the wound healing process. We found that patients who can achieve intensive glycemic levels during hospitalization and can maintain stable blood glucose levels after discharge show rapid wound healing and improved prognosis.

Several studies have confirmed that many miRNAs participate in the process of wound healing [26–29]. During the inflammatory period of the wound, miRNA-181c and miRNA-21-5p promote the expression of M2 macrophages to inhibit the inflammatory response and shorten the inflammatory response time [30, 31]. The mechanism might involve miRNAs carried by exosomes that inhibit toll-likereceptor-4 activity, thereby promoting the differentiation of M1 macrophages into M2 macrophages and inhibiting the production of inflammatory factors. This process accelerates the development of the inflammatory response phase into the proliferative phase [32]. The proliferative phase of wound healing mainly involves neovascularization and the proliferation of fibroblasts. Some studies have demonstrated that exosomes, miR-126-3p, miRNA-21-5p, and miRNA-152-3p, derived from mesenchymal stem cells, regulate the protein kinase (ERK) signaling pathway, the phosphatidylinositol 3-kinase/protein kinase B (PI3K/AKT) pathway, and the signal transducer and activator of transcription 3 (STAT-3) pathway that promote the formation of peripheral neovascularization [33, 34].

In this study, serum exosomal miR-126-3p and miR-125b-1-3p levels increased after glycemic control treatment, which was positively correlated with the wound healing rate (Figures 3(a)–3(d)). The serum exosomal miR-126-3p level in the intensive glycemic control group was significantly higher than that in the conventional glycemic control group (Figure 4). These findings were consistent with those from previous studies [35], and statistical analysis revealed that serum exosomal miR-126-3p promotes wound healing. This experiment found that intensive glycemic control increases the serum exosomal miR-126-3p level in patients with diabetic ulcers and promotes wound healing. The underlying mechanism may be related to serum exosomal miR-126-3p, which promotes endothelial cell migration and peripheral neovascularization formation [36]. However, the specific mechanism needs to be elucidated further. There are also some deficiencies in this experiment. The sample size of the experiment was small, and the follow-up sample size needs to be increased to further confirm the correlation between miR-126-3p and diabetic ulcer wound healing and to explore its mechanism of action in regulating wound healing.

In summary, we found that the levels of serum exosomes, namely, miR-125b-1-3p and miR-126-3p, are correlated with wound healing in diabetic ulcers. Intensive glycemic control can increase the serum exosome miR-126-3p level in patients with diabetic ulcers and promote wound healing. This study provides new ideas for exploring the mechanism by which intensive glycemic control promotes wound healing. Our team will also further explore the targets of action based on this experiment.

## Figures and Tables

**Table 1:** qRT-PCR primers.

Genes	Primer sequence (5′-3′)
miR-125b-1-3p	ACGGGTTAGGCTCTTGGGA
miR-126-3p	TCGTACCGTGAGTAATAATGCG
U6	CTCGCTTCGGCAGCACA

**Table 2:** Baseline characteristics of two groups ($\bar{x}\pm s$).

Characteristics	Intensive glycemic control group (*n* = 21)	Conventional glycemic control (*n* = 24)	Test value	*P* value
Male (*n*, %)	14 (66.7%)	20 (83.3%)	-	1.000
Age (years)	58.00 ± 8.96	57.88 ± 9.72	0.05	0.97
BMI (kg/cm^2^)	23.40 ± 4.52	23.58 ± 3.92	0.15	0.88
FBG (mmol/L)	10.43 ± 3.82	10.64 ± 2.86	0.21	0.83
2 h PBG (mmol/L)	13.91 ± 3.18	13.51 ± 2.76	0.45	0.66
SYS (mmHg)	132.24 ± 29.70	138.25 ± 21.68	0.78	0.44
DIA (mmHg)	81.90 ± 16.69	80.67 ± 12.41	0.29	0.78
HbA1c (%)	10.76 ± 4.51	10.54 ± 3.55	0.18	0.86
Albumin (g/L)	32.22 ± 6.63	33.82 ± 6.71	0.80	0.43
Globulin (g/)	30.81 ± 6.26	29.72 ± 7.09	0.54	0.59
Blood creatinine (*μ*mol/L)	101.55 ± 46.05	86.76 ± 26.78	0.17	0.86
Blood uric acid (*μ*mol/L)	277.00 ± 79.47	303.31 ± 100.47	1.12	0.27
Cholesterol (mmol/L)	3.98 ± 1.16	4.30 ± 1.54	0.79	0.43
Triglycerides (mmol/L)	1.37 ± 0.72	1.82 ± 0.71	1.99	0.06
LDL-c (mmol/L)	2.36 ± 1.00	3.09 ± 1.14	1.16	0.25
WBC (×10^9^/L)	11.13 ± 4.37	8.88 ± 2.01	1.57	0.13
Percentage of neutrophils (%)	72.81 ± 18.18	71.44 ± 12.69	0.08	0.94
Hb (g/L)	113.06 ± 21.42	120.95 ± 22.40	1.13	0.27

- indicates that there is no corresponding statistical value. BMI, body mass index; FBG, fasting blood glucose; 2 h PBG, 2-hour postprandial blood glucose; SYS, systolic pressure; DIA, diastolic pressure; HbA1c, glycosylated hemoglobin A1c, LDL-c, low-density lipoprotein cholesterin; WBC, white blood cell; Hb, hemoglobin.

**Table 3:** Comparison of clinical data within and between groups ($\bar{x}\pm s$) or *M* (*Q*_*L*_ ~ *Q*_*u*_).

Characteristics	Intensive glycemic control group (*n* = 21)			Conventional glycemic control (*n* = 24)		
	Before treatment	After treatment	Difference before and after treatment	Before treatment	After treatment	Difference before and after treatment
FBG (mmol/L)	10.43 ± 3.82	5.49 ± 0.64^*∗∗∗*^	3.90 (2.15, 7.70)	10.64 ± 2.86	7.97 ± 1.76^*∗∗∗*^^###^	1.95 (1.00, 2.70)^▲###^
2 h PBG (mmol/L)	13.91 ± 3.18	7.53 ± 1.74^*∗∗∗*^	4.80 (3.90, 9.15)	13.51 ± 2.76	10.72 ± 1.86^*∗∗∗*^^###^	2.55 (−0.70, 5.13)^▲###^
SYS (mmHg)	132.24 ± 29.70	124.33 ± 21.27	3.00 (−5.00, 26.00)	138.25 ± 21.68	128.88 ± 17.43^*∗∗*^	5.00 (1.00, 11.50)^▲^
DIA (mmHg)	81.90 ± 16.69	76.62 ± 13.00	−6.00 (−14.00, 10.00)	80.67 ± 12.41	78.63 ± 11.00	−1.00 (−3.00, 3.75)^▲^
Albumin (g/L)	30.54 ± 6.68	32.53 ± 6.51	0.60 (−2.30, 4.50)	33.82 ± 6.71	35.25 ± 5.82^*∗*^	0.50 (0.00, 6.15)^▲^
Blood (*μ*mol/L)	101.55 ± 46.05	99.82 ± 42.73	−1.00 (−12.00, 10.25)	86.76 ± 26.78	82.65 ± 25.94	−0.50 (−11.00, 9.75)^▲^
Blood uric acid (*μ*mol/L)	277.00 ± 79.47	285.45 ± 104.645	16.00 (−15.00, 23.00)	303.31 ± 100.47	290.44 ± 106.11	−17.00 (−89.25, 74.50)^▲^
Hb (g/L)	113.06 ± 21.42	103.89 ± 24.30^*∗*^	−6.50 (−16.50, 0.50)	120.95 ± 22.40	111.68 ± 26.07^*∗∗*^	−14.50 (−22.75, 3.50)^▲^
WBC (×10^9^/L)	11.13 ± 4.37	7.34 ± 3.22^*∗∗*^	−3.59 (−8.51, 0.31)	8.88 ± 2.01	8.09 ± 3.61	−0.70 (−2.37, 0.83)^▲#^
Percentage of neutrophils (%)	72.81 ± 18.1	63.21 ± 13.01^*∗*^	−11.25 (−20.35, −5.08)	71.44 ± 12.69	65.45 ± 11.66^*∗*^	−4.95 (−13.20, 0.20)^▲^
Wound (cm^2^)	5.60 (1.87, 20.73)	2.22 (0.54, 5.85)^△^^*∗∗∗*^	61.55 ± 21.54	8.85 (1.99, 21.31)	6.49 (1.13, 14.09)^△^^*∗∗∗*^^▲###^	35.70 ± 15.74^###^

In the column of wound area, the difference before and after treatment is the wound healing rate. Wound healing rate = (original wound area − unhealed wound area)/original wound area × 100%. ^△^Wilcoxon rank-sum test, ^▲^Mann–Whitney *U* test. Intragroup comparison, ^*∗*^*P* < 0.05, ^*∗∗*^*P* < 0.01, and ^*∗∗∗*^*P* < 0.001. Comparison between groups (including the difference before and after treatment), ^#^*P* < 0.05, and ^###^*P*  <  0.001.

**Table 4:** The prognosis of two groups.

Prognosis	Intensive glycemic control (*n* = 19)	Conventional glycemic control group (*n* = 22)	*Z*	*P*
Good/bad	13/6	8/14	−2.02	<0.05

## Data Availability

The data in the manuscript are from clinical laboratory collection and experiment results. More detailed supporting data can be provided upon request to the corresponding author.
